# Accoustic immitance measures in infants with 226 and 1000 hz probes: correlation with otoacoustic emissions and otoscopy examination

**DOI:** 10.1016/S1808-8694(15)30836-3

**Published:** 2015-10-18

**Authors:** Michele Vargas Garcia, Marisa Frasson de Azevedo, José Ricardo Testa

**Affiliations:** 1 Specialist, Speech and Hearing Therapist.; 2 PhD, Adjunct Professor - Universidade Federal de São Paulo-UNIFESP/ Escola Paulista de Medicina.; 3 PhD, Adjunct Professor of Otorhinolaryngology - Universidade Federal de São Paulo/UNIFESP/Escola Paulista de Medicina/EPM.

**Keywords:** hearing, child, middle ear, acoustic impedance tests

## Abstract

Audiological evaluation in infants should include the middle ear (immitance measures and otoscopy) and also a cochlear evaluation. **Aim:** To check which tympanometry tone test (226 Hz or 1000 Hz), transient otoacoustic emissions and otoscopy. **Methods:** Transient otoacoustic emissions were taken from sixty infants ranging from zero to four months of age. The babies were assigned to two groups of 30 infants each, according to the presence or absence of otoacoustic emissions (OAE). All babies have undergone tympanometry with probe tones of 226 and 1000 Hz and ENT evaluation. **Results:** Tests performed with 1000 Hz probe tone were more sensitive in identifying middle ear disorders. In children with normal tympanograms, both probe tones (226 and 1000 Hz) showed high specificity. All correlations were significant when the 1000 Hz probe tone was used. **Conclusion:** The high frequency probe tone (1000 Hz) presented the most significant correlation with OAE and otoscopy in infants from zero to four months of age.

## INTRODUCTION

The early diagnosis of hearing impairment (HI) in children must enjoy special attention from health care professionals, especially from pediatricians, otolaryngologists and speech and hearing therapists.

In order to have a trustworthy audiologic diagnosis in infants, it is necessary to assess the middle ear conditions, because they can cause temporary conductive hearing loss and impact cochlear function studies.

In order to assess cochlear function (outer hair cell integrity), the infants are submitted to Evoked Otoacoustic Emissions recording and analysis, and the transient click stimulus (TOAE) is the one recommended for neonatal auditory screening^1^, ^2^.

Pressure variations in the external auditory canal and/or in the middle ear impact the amplitude, spectrum and reproducibility of Evoked Otoacoustic Emission responses^3^.

When the newborn does not respond to the Otoacoustic Emissions test, it is necessary to submit him/her to an otolaryngological evaluation in order to look for alterations in the external auditory canal and/or the middle ear. Together with medical evaluation, it is necessary to assess acoustic immittance values in order to assess the infant’s middle ear conditions.

Acoustic immittance measures contribute with information about middle ear mobility and the auditory pathway integrity at this level. They are very much used in clinical practice with infants for being an objective evaluation providing the tympanometric curve and the acoustic reflexes.

Conventional tympanometry is carried out with the 226Hz test tone and the results with this tone have considerable diagnostic value for elderly, adult and pediatric patients starting at 6 years of age; however, in relation to newborns and infants, there are controversies. Studies have shown that infants without OAE can have a normal tympanometric curve at the study with 226Hz test tone, even when there are conductive alterations. Thus, the application of the highest test tone (1,000Hz) has been suggested by some authors, because mild middle ear problems would not be detected by the 226Hz^4^, ^5^ probe.

On the other hand, in studies carried out in Brazil, Carvallo^6^ and Linares^7^ advocated the use of the 226Hz probe in children from 0 to 8 months, since they found matching results in their assessments.

Starting from the aforementioned considerations, we can stress that it is very important that the tympanometric curve be obtained with accuracy. Thus justifying the need to study tympanometric curves by means of two test-tones (226Hz and 1000Hz) and check if there is any difference in the tympanometric responses in relation to tones, as well as doing a joint analysis of the Transitory Otoacoustic Emission Test and otolaryngological medical evaluation.

Thus, in this study we aim at checking which test tone for tympanometry (226Hz or 1000Hz) is more correlated with the otorhinolaryngology evaluation and the results from the Otoacoustic Emissions by transient stimulus in infants from zero to four months.

## MATERIALS AND METHOD

This study was approved by the Ethics in Research Committee, under protocol # 0723/06.

Following ethic principles of research with human beings, the parents and/or guardians agreed with their children’s participation in this study and signed the free and informed consent form.

The sample was made up of 60 infants, of both genders, of an age range from zero to four months, distributed in two groups. Group I: Thirty infants with Transient Otoacoustic Emissions and Group II: thirty infants without Transient Otoacoustic Emissions.

In order to make up the groups, the infants had to be between zero and four months, with and without risk indicator for hearing impairment. We ruled out all the infants with external acoustic canal malformation, since it would make it impossible to evaluate them in this study, as well as infants with neurological alterations and/or genetic syndromes.

Each evaluation was carried out following this study’s protocol. This study was considered a double blind, since the examiners were not aware of results from the other tests the infants were submitted to. The otorhinolaryngologist did not know to which group the infant belonged to, and the researcher was not aware of the medical evaluation results and only had access to all the results after the conclusion of the exams the infants were submitted to. All the evaluations were carried out on the same date. The parameters considered in this study were the following:

1. Otoscopic exam: the infants were assessed by the otorhinolaryngologist for otoscopy, to check the conditions of the external acoustic meatus and the tympanic membrane. For this study, the tympanic membrane conditions were considered, and were classified as normal or altered (retracted hyperemic, opaque, perforated, and bulged). The physician in charge of the evaluation has more than fifteen years of experience with newborns.
2. Recording and analysis of the Otoacoustic Emissions by Transient Stimulus (TOAE): The infants were submitted to Transient Otoacoustic Emissions recording and analysis, considering Finitzo’s criteria (1998) recommended by Chapchap (1996)^8^ and Azevedo (2003)^9^, and they were: click stimulus, with 75-83 dBpeSPL stimulus intensity, in the frequency range between 1,500 and 4,000Hz. TOAE was considered present when the signal/ noise ratio by frequency band was ≥ 3 dB for 1,500Hz and ≥ 6 dB for 2,000Hz, 3,000 and 4,000Hz and the general reproducibility considered was ≥50% and the probe stability ≥70%. In the absence of these responses, the infant did not have otoacoustic emissions. The transient stimuli otoacoustic emissions were carried out with the infants inside a sound treated booth. The equipment used was the ILO 96-Otoacoustic Emissions Analyzer, coupled to a microcomputer, using the “Quickscreener”.^3^. Acoustic Immittance Measures: tympanometry was carried out in the infants by means of a Middle Ear Analyzer: Impedance Audiometer- AT235h- Interacoustics. The tympanometric curve was carried out by the 226Hz and the 1000Hz test tones. The tympanometry was captured in two frequencies in order to observe whether there would be a difference in the infants’ tympanometric curves, and the probe suggested in the literature to assess this age range is the 1000Hz probe. The tympanometric curves were classified according to Jerger (1970)^10^ and Carvallo (1992)^6^ in: Type A - a single admittance peak between -150 and +100 daPa and 0.2 to 1.8ml volume; Type C - Admittance peak shifted towards the negative pressure side; type D - Double peak curve; asymmetrical curve - peak at high positive pressure; inverted curve - with inverted shape in relation to the normal curve and B-type flat curve - without an admittance peak. The statistical analyses were carried out by means of the chi-square test.

## RESULTS

The results are being presented by ear (right and left) and by group (emissions present and absent). Firstly we analyzed the tympanometry findings with the 226Hz test tone in relation to the otoscopic evaluation, considering tympanic membrane conditions. Tables 1 and 2 show the correlations between the tympanometry findings with the 226Hz test tone and the otoscopic evaluation for both ears.

**Table 1:** Tympanometry with the 226Hz test tone and right ear otoscopic evaluation in both groups.

ENT TM RE							
	Tymp RE 226	Altered		Normal		Total	
		Qtity	%	Qtity	%	Qtity	%
	Altered	1	11,1%	2	9,5%	3	10%
Group I	Normal	8	88,9%	19	90,5%	27	90%
	Total	9	30,0%	21	70,0%	30	100%
p- value	0,894						
	Altered	2	11,1%	1	8,3%	3	10%
Group II	Normal	16	88,9%	11	91,7%	27	90%
	Total	18	60,0%	12	40,0%	30	100%
p- value	0,804						

significant p-value < 0.05 (5%). Legend: group I: infants with otoacoustic emissions, group II: infants without otoacoustic emissions; RE: right ear; TM: tympanic membrane, Tymp: tympanometry; ENT: otolaryngology; Qtity: Quantity

**Table 2:** Tympanometry with the 226Hz tone test and left ear otoscopic evaluation in both groups.

ENT TM LE							
	Tymp LE 226	Altered		Normal		Total	
		Qtity	%	Qtity	%	Qtity	%
	Altered	2	18,2%	1	5,3%	3	10%
Group I	Normal	9	81,8%	18	94,7%	27	90%
	Total	11	36,7%	19	63,3%	30	100%
p- value	0,256						
	Altered	1	14,3%	5	21,7%	6	20%
Group II	Normal	6	85,7%	18	78,3%	24	80%
	Total	7	23,3%	23	76,7%	30	100%
p- value	0,333						

significant p-value < 0.05 (5%). Legend: group I: infants with otoacoustic emissions, group II: infants without otoacoustic emissions; LE: Left ear; TM: tympanic membrane, Tymp: tympanometry; ENT: otolaryngologist. Qtity: Quantity

We did not find any statistically significant correlation between the otoscopic evaluation and the tympanometry findings with the 226Hz tone test.

We analyzed the tympanometry findings with the 1000Hz test tone in relation to the otoscopic evaluation (tympanic membrane conditions) in both groups, which is shown on Tables 3 and 4.

**Table 3:** Tympanometry with the 1000Hz test tone and right ear otoscopic evaluation in both groups.

ENT TM RE							
	Tymp RE 1000	Altered		Normal		Total	
		Qtity	%	Qtity	%	Qtity	%
	Altered	1	11,1%	2	9,5%	3	10%
Group I	Normal	8	88,9%	19	90,5%	27	90%
	Total	9	30,0%	21	70,0%	30	100%
p- value	0,894						
	Altered	17	94,4%	7	58,3%	24	80%
Group II	Normal	1	5,6%	5	41,7%	6	20%
	Total	18	60,0%	12	40,0%	30	100%
p- value	0,015*						

* significant p-value < 0.05 (5%) Legend: group I: infants with otoacoustic emissions, group II: infants without otoacoustic emissions RE: right ear; TM: tympanic membrane, Tymp: tympanometry; ENT: otolaryngologist

**Table 4:** Tympanometry with 1000Hz test tone and left ear otoscopic evaluation in both groups.

ENT TM LE							
	Timp OE 1000	Altered		Normal		Total	
		Qtity	%	Qtity	%	Qtity	%
	Altered	4	36,4%	2	10,5%	6	20%
Group I	Normal	7	63,6%	17	89,5%	24	80%
	Total	11	36,7%	19	63,3%	30	100%
p- value	0,804						
	Altered	19	95,0%	4	40,0%	23	77%
Group II	Normal	1	5,0%	6	60,0%	7	23%
	Total	20	66,7%	10	33,3%	30	100%
p- value	0,001*						

* significant p-value < 0.05 (5%). Legend: group I: infants with otoacoustic emissions, group II: infants without otoacoustic emissions; LE: left ear; TM: tympanic membrane, Tymp: tympanometry; ENT: otolaryngologist. Qtity: Quantity

There was a statistically significant difference in group II both for evaluations with alterations (considering a retracted tympanic membrane, hyperemic, opaque, perforated, bulged and/or B or C tympanometric curve), as well as for evaluations within normal values in relation to the 1000Hz test tone when compared to the otoscopic evaluation. We did not find any perforated tympanic membrane.

**Chart 1:**
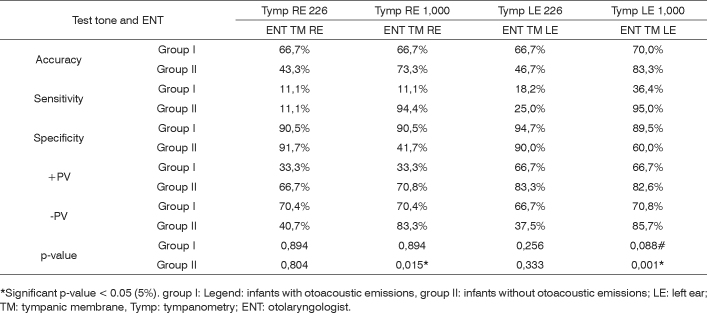
Summary of the descriptive values: specificity, sensitivity, accuracy, false positive, false negative and p-value (chi-squared) in relation to the otorhinolaryngological assessment and multiple frequencies tympanometry.

Following, we present a Chart (Chart 1) summarizing the descriptive measures: specificity, sensitivity, accuracy, false positive, false negative and p-value (chi-squared).

*Significant p-value < 0.05 (5%). group I: Legend: infants with otoacoustic emissions, group II: infants without otoacoustic emissions; LE: left ear; TM: tympanic membrane, Tymp: tympanometry; ENT: otolaryngologist.

It was possible to see that the 1,000Hz test tone in tympanometry was more sensitive in Group II and more specific in Group I, and the 226Hz test tone was more specific for groups I and II.

We tried to see which tympanometry tone test (226Hz or 1,000Hz) has a greater correlation with the otoacoustic emissions in infants in order to observe the type of tympanometric curve in each test tone for each group studied, in order to facilitate the diagnosis of conductive hearing disorders, especially in group II. If the professional is aware of this correlation, he/she can be more efficient in referring the patient to a differential diagnosis and it enhances the speed of the audiologic diagnostic.

Tables 5 and 6 showed the tympanometry findings and their correlations with the 226Hz test tone and otoacoustic emissions for both ears in both groups.

**Table 5:** Tympanometry with the 226Hz test tone and the otoacoustic emissions in the right ear for both groups.

Tymp RE 226						
	Altered		Normal		Total	
	Qtity	%	Qtity	%	Qtity	%
Group I	3	50,0%	27	50,0%	30	50%
p-value	1,000					
Group II	3	50,0%	27	50,0%	30	50%
p-value	1,000					

Total	6	10,0%	54	90,0%	60	100%

Significant p-value < 0.05 (5%) Legend: group I: infants with otoacoustic emissions; group II: infants without otoacoustic emissions; RE: right ear; TM tympanic membrane; Tymp: tympanometry; ENT: otolaryngologist; Qtity: Quantity

**Table 6:** Tympanometry with the 226hz test tone and otoacoustic emissions in the left ear from both groups.

Timp OE 226						
	Altered		Normal		Total	
	Qtity	%	Qtity	%	Qtity	%
Group I	3	33,3%	27	52,9%	30	50%
p-value	0,278					
Group II	6	66,7%	24	47,1%	30	50%
p-value	0,278					

Total	9	15,0%	51	85,0%	60	100%

Significant p-value < 0.05 (5%) Legend: group I: infants with otoacoustic emissions; group II: infants without otoacoustic emissions; LE: left ear; TM tympanic membrane; Tymp: tympanometry; ENT: otolaryngologist; Qtity: Quantity

We did not see statistically significant differences in the correlation between otoacoustic emissions and the 226Hz probe tympanometry, both for altered and normal patients from the two groups.

The correlations between the 1,000Hz tympanometry and the otoacoustic emissions are presented on Tables 7 and 8.

**Table 7:** Tympanometry with the 1,000Hz test tone and otoacoustic emissions in the right ear for both groups.

Tymp RE 1000						
	Altered		Normal		Total	
	Qtity	%	Qtity	%	Qtity	%
Group I	3	11,1%	27	81,8%	30	50%
p-value	<0,001*					
Group II	24	88,9%	6	18,2%	30	50%
p-value	<0,001*					

Total	27	45,0%	33	55,0%	60	100%

* Significant p-value < 0.05 (5%) Legend: group I: infants with otoacoustic emissions; group II: infants without otoacoustic emissions; RE: right ear; TM tympanic membrane; Tymp: tympanometry; ENT: otolaryngologist; Qtity: Quantity

**Table 8:** Tympanometry with the 1,000Hz test tone and otoacoustic emissions in the left ear for both groups.

Timp OE 1000						
	Altered		Normal		Total	
	Qtity	%	Qtity	%	Qtity	%
Group I	6	20,7%	24	77,4%	30	50%
p-value	<0,001*					
Group II	23	79,3%	7	22,6%	30	50%
p-value	<0,001*					

Total	29	48,3%	31	51,7%	60	100%

* Significant p-value < 0.05 (5%) Legend: group I: infants with otoacoustic emissions; group II: infants without otoacoustic emissions; LE: left ear; TM tympanic membrane; Tymp: tympanometry; ENT: otolaryngologist; Qtity: Quantity

We did find a statistically significant correlation for the 1,000Hz test tone and the otoacoustic emissions both for infants with abnormalities in their evaluations as well as those who were found normal in both groups.

**Chart 2:**
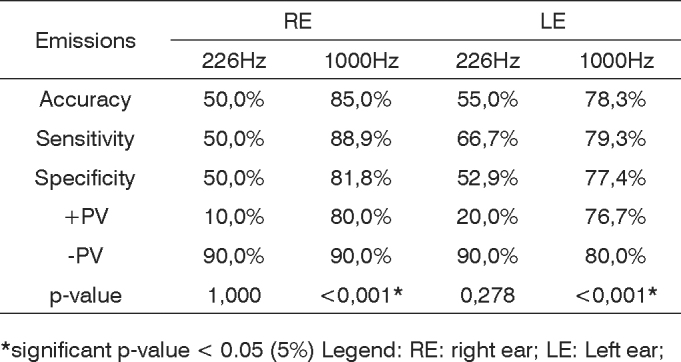
Accuracy, sensitivity and specificity values obtained from the correlation between the multiple frequency tympanometry and the otoacoustic emissions.

Following we see a chart (Chart 2) with the values for accuracy, sensitivity and specificity obtained from the correlation between the multiple frequency tympanometries and the otoacoustic emissions.

*significant p-value < 0.05 (5%) Legend: RE: right ear; LE: Left ear;

We notice that for the 1,000Hz test tone there is a statistically significant relationship between the tympanometry curve and the otoacoustic emissions, and this relation is valid for both ears from both groups. When we compare the tympanometry test tone with the otoacoustic emissions, the 1,000Hz test tone has a high sensitivity and specificity percentage.

## DISCUSSION

The detection and follow up of otologic diseases are paramount, especially in the first months of life. In the pediatric otolaryngological practice, the identification of children with acute disorders and febrile and/or painful manifestations is among the most frequent problems.

All the results available must be used in an attempt to detect these alterations. The otologic evaluation of middle ear dysfunctions in infants is more accurate when added to otorhinolaryngological evaluation and immittance tests.

Infant’s middle ears can have otitis because of numerous causes and, according to Paparella^11^, if well taken care of, they do not leave sequelae; however, if left untreated it can become a chronic disease. According to Ingvarsson et al.^12^ and Santos^13^ it is one of the frequent problems that most happen to children.

Having these quotations in mind, it is very important to accurately diagnose otitis media cases and the combination of an otorhinolaryngological evaluation and tympanometries represent an efficacious and feasible combination. The Joint Committee on Infant Hearing^14^ suggests that acoustic immittance in infants must be part of the audiologic battery of tests. Purdy, Willians^15^ mentions the need to better use tympanometry in infants.

Otoscopic evaluation in infants must be carried out by an experienced physician, because the external acoustic meatus in very small, thus making it difficult to see the tympanic membrane. Besides experience, it is necessary to have a good otoscope that enhances view. In the present investigation, the otoscopic evaluations met these needs. In order to carry out the tympanometry, all the infants were in a light sleep and the procedure was carried out in a fast and careful way in order to properly seal the external auditory canal, as recommended by Carvallo^16^.

In this study, one of the goals was to correlate the multiple frequencies tympanometry with the otoscopic evaluations in order to check for results’ reliability as well as to observe the sensitivity and specificity of the tests employed.

In our study, there was no statistically significant correlation between the otoscopic evaluation and the 226Hz test tone tympanometry findings (Tables 1 and 2). When the otoscopic evaluation presented some alteration, the 226Hz test tone was within normal limits. The statistical analyses were carried out by ear, thus, for the right ears in group I, 88.9% of the tympanometric assessments were normal with altered otoscopic evaluation (Table 1). For the left ear, in the same group, 81.8% of the ears had normal tympanometry exams and had altered otoscopic evaluation (Table 2). Group II also had high percentages of normal tympanometric curves and alterations seen in the otoscopic evaluation, 88.9% for the right ear and 85.6% for the left ear (Tables 1 and 2).

The 1000Hz test tone was correlated with the otoscopic evaluation, we found a statistically significant difference in group II, both for the altered evaluations (considering the tympanic membrane retracted, hyperemic, opaque, perforated, bulged and/or B and C tympanometric curve) as well as for the normal evaluations (Tables 3 and 4). In this group, for the right ear, 94.4% of the ears were altered in both evaluations (Table 3) and regarding the left ear, 95% of the ears also had alterations (Table 4).

The tests’ sensitivity and specificity were checked by means of statistical tests. It was possible to observe that the 226Hz test tone has high specificity percentage for both groups I and II, being around 90% in both groups for both ears. Thus, this test tone is able to pick those evaluations which are within normal limits. The 1,000Hz tone test had high sensitivity (94.4% for the right ear and 95% for the left ear) in group II, and it was appropriate to identify those altered evaluations. It was also specific for Group I (90.5% for the right ear and 89.5% for the left ear (Chart 1)).

Between the two study groups, we assessed 60 infants, 31 (51.6%) had altered bilateral otoscopy and tympanometry with the 1,000Hz test tone, 24 (40%) infants had bilateral alteration and 8 (14%) had unilateral alteration, adding up to 54% of infants with altered tympanograms. Thus, it is possible to see the proximity of alterations picked by the otoscopic evaluation and the 1,000Hz test tone tympanometry (51.6% and 54%) reinforcing the validity of both tests for this diagnosis.

Cone-Wesson et al.^17^ carried out a study with a group of infants between 8 and 12 months. They used the 226Hz test tone and found 58% of infants with middle ear alterations. In the present investigation, the higher percentages of alterations were found with the 1,000Hz test tone, similar to what was found by the author. The sample’s age range is different, as are the test tones used, but the number of children with alterations matched ours (54%).

The 1,000Hz test tone use indications in infant tympanometry come from anatomical and physiological differences in the middle ear, as described by Holte et al.^18^, Moore^19^.

Margolis, Hunter^20^ stressed that the mass components are higher in high frequency waves (such as the 1,000Hz, for example) and the lower in the low frequency probes (as the 226Hz, for example). These statements help to justify the findings of the present investigation.

The 1,000Hz tympanometry and the otorhinolaryngological evaluation were sensitive to identify middle ear alterations and this finding exists in many studies (Franche et al.^21^; Purdy et al.^22^, Sutton et al.)^23^. Margolis^3^ also advocates the routine use of tympanometry with the 1,000Hz test tone to assess infants’ middle ears.

In this study, Group II had 23 (76% of the group) infants with bilateral alteration in their tympanometric curve with the 1,000Hz test tone. Campbell^25^ stresses the importance of diagnosing conductive hearing alterations so as not to delay the diagnosis of conductive and cochlear pathologies. In this study, only one infant (4%) from Group II was diagnosed as having a cochlear alteration, and this diagnosis happened early on, thus yielding treatment at the proper time.

All the infants diagnosed with middle ear alterations by means of the otoscopic evaluation and tympanometry received immediate medical treatment and were reassessed within 10 days. Thus, it was possible to have early diagnosis and treatment, avoiding a possible diagnostic delay, as mentioned by Campbell^24^.

In terms of the type of otorhinolaryngological alteration found, in Group I (infants with OAE present), 66.6% of the ears were within the normal range, 1.7% with ear drum retraction; 1.7% with hyperemia and 30% with opacity. In Group II, 40% of the infants had normal evaluations, 3.3% had retracted tympanic membranes, none had hyperemia and 56.7% had opacity of the ear drum. It was then possible to observe that in Group II (infants without OAEs) there were more cases of tympanic membrane opacity. In a study carried out by Saeed et al.^25^ they performed tympanometry and otoscopy in children with otitis media with effusion and concluded that they were sensitive for the diagnosis of middle ear effusion during acute otitis media. These findings are in agreement with those from the present study, since the middle ear alteration was identified. In the current study there were no infants with middle ear effusion, but they had opacity, retraction and hyperemia. They were all treated and followed up by the physician.

Saes et al.^26^ studied 195 children from zero to two years of age to investigate middle ear alterations. The children were submitted to otoscopy and tympanometry. They found that 68.4% of the infants had one or more episodes of middle ear effusion in their first two years of life and the age at which this was more prevalent was between four and 12 months. In this study’s sample, all the infants had between zero and six months of age, being within the period mentioned in the study above, which found more middle ear alterations. The findings from the present study agree with those from Saes et al.^26^, since 76% of the infants from Group II had tympanometry alteration (Table 8) and 63.4% of the infants from the same group had altered otorhinolaryngological exam.

In a study led by Rhode^27^ there was a greater correlation between the 1,000Hz tympanometry and OAE and BEAP, however, not with otoscopy. There were more alterations seen at otoscopy (43% of the sample’s ears) than at the tympanometry (1% of the sample’s ears), thus, there was no good correlation among the assessments. These results differ from those of the present study, because the correlation between the otoscopy and the 1,000Hz tympanometry was significant (Tables 3 and 4).

Capellini^28^ states that it is not necessary to use the high tone test with infants, because he did the study with the 226Hz tone and found results matching those from the otorhinolaryngological exam for normal infants from zero to six months of age. The author’s finding are in agreement with those from the present study, because the specificity found for the 226Hz tone in normal infants was higher than 90% (Chart 1). Thus, the 226Hz test tone tympanometry is able to accurately analyze infants with normal middle ears.

The analyses of the methods used to carry out the tympanometry is paramount for the speech and hearing therapist to be sure about obtaining results, because the tympanometric curve difference interferes in the type of hearing loss the infant may have. The middle ear immittance study offers a large number of practical diagnostic applications, such as, for example, information about the functional integrity of the tympanic-ossicular system.

Middle ear mechanic-acoustic properties in newborns must be studied, because correlations with evoked otoacoustic emissions are important to speed up the diagnosis of conductive hearing loss. Tympanometric studies with infants below six months of age have not been broadly carried out and are necessary in order to enhance the use of tympanometry in the auditory diagnosis at this age range.

According with Northern and Downs^29^, middle and outer ear structures change with the child’s development, becoming more like the adult ones at the age of nine years.

In the present study, one of the goals was to check the relationship between multiple frequency tympanometry and the OAEs, in order to analyze which had the best correlation.

In the sample studied here, there was no statistical significant difference in the correlation between otoacoustic emissions and the 226Hz tympanometry, both for altered cases as well as for the normal patients in both groups (Tables 5 and 6). Both for Group I and Group II, we noticed that, for the right ear, 50% of the infants’ ears had normal tympanometric curves, and this was not statistically significant (Table 5). The same happened to the left ear, in Group I, 52.9% were normal and for Group II, 47.1% were normal (Table 6). Thus, the 226Hz test tone did not present significant data for this correlation in the two groups studied.

There was a statistically significant correlation for the 1,000Hz test tone and the otoacoustic emissions for infants with abnormalities in the exams as well as for those who had normal results in both groups (Tables 7 and 8). For the right ear of Group I infants, we observed that 81.8% of the tympanometries were normal, and for those in Group II, 88.9% had alterations (Table 7). As to the left ear, for Group I, 77.4% of the tympanometries were normal and for Group II, 79.3% of the tympanometries were altered (Table 22).

Silva30 and Callandrucio et al.^31^ carried out a study with infants using OAEs and the 1,000Hz test tone tympanometry and found good correlations between the tests, and the same was found in this study’s sample.

Vartiainem32 and Keefe et al.^33^ mention that the otoacoustic emissions are very sensitive to middle ear alterations, and this is in agreement with the findings of our study. In the current investigation, of the 30 infants evaluated in Group II, 29 (96.6%) had uni or bilateral disease, and thus failed the OAEs. Only 1 (4.4%) infant was diagnosed with sensorineural hearing loss by the BAEP, after treatment of the conductive hearing loss.

In Group I, the 30 infants had OAEs, and 22 (73% of the group) had normal tympanometric curve, with the 1,000Hz test tone being normal in both ears; 1 (0.03% of the group) bilaterally altered and 7 (20% of the group) with unilateral alteration. Even with an altered tympanometric curve, the infants passed the OAEs test. It is believed that this middle ear disease could have been in an initial or final stage, and this did not interfere in the OAEs results. This finding agrees with the one stated by Ameed (1995)34 who says that the presence or absence of OAEs is associated with the type of fluid present in the middle ear, and effusion with mucous is the one that has the greatest likelihood of failing TOAE.

In this study, the conductive alterations were precisely diagnosed with the 1,000Hz tone test tympanometry, thus, the failure of otoacoustic emissions in Group II was associated with this alteration, and this confirmed that it is not a cochlear alteration.

The findings from this study are in agreement with the statement from Carvallo, Ravagnani and Sanches^35^ who say the combined application of the acoustic immittance and otoacoustic emissions measures can clarify issues regarding the level of middle ear involvement which prevent OAEs capture.

Studies such as the one from Zapala^36^ and Koivunem et al.^37^ correlated the OAEs with multiple frequency tympanometries in newborns in order to check for conductive hearing loss. In the present investigation, there is an agreement with the findings of the aforementioned authors, since they report greater reliability with the 1,000Hz test tone in tympanometries performed with the OAEs. Sutton et al.^23^, McKinley et al.^38^ Margolis^3^ believe this correlation between OAEs and the 1,000Hz test tone tympanometry is good.

The study by Soares^39^ is in agreement with the findings of the present investigation, because they also found a good correlation between OAEs and the 226Hz tympanometry for normal infants of the same age range. In the current study, the 226Hz test tone had high specificity (Chart 1), being reliable to identify normal evaluations.

The findings of the present investigation are in agreement with those from Rhodes et al.^27^ who compared the 226Hz, 678Hz and 1,000Hz tympanometries, with the results from the otoacoustic emissions and the BAEP and found a greater correlation with the high frequency tympanometry.

In the present study, 8% of the Group II infants (without OAE) had type C tympanometric curve with the 1,000Hz test tone, indicating negative pressure in the middle ear. This finding is in agreement with those from Marshall et al.^40^ who reported that small quantities of middle ear negative pressure could affect OAEs’ amplitude and spectrum.

For this correlation studied (OAEs and acoustic immittance values), the 1,000Hz test tone sensitivity was of 88.9% for the right ear and 79.3% for the left ear. The specificity of this same tone was of 81.8% for the right ear and 77.4% for the left ear (Chart 2). Studies like the ones led by Himelfarb et al.^41^ have already proven that the higher test tone for infant tympanometry would capture more alterations than the lower tone, establishing more sensitivity. These findings as to the sensitivity of the 1,000Hz test tone agree with other studies by Hunter & Margolis^42^, Willians et al.^43^, Keefe & Levi^4^.

Ho et al.^44^ reported on the difficulties researchers have to differentiate the conductive from the cochlear disorder when the infant fails the OAE test. This was not seen in the present investigation because we used the 1,000Hz test tone tympanometry, which was sensitive enough to identify middle ear alterations together with the otolaryngological alteration.

## CONCLUSION

The 1,000Hz tone test showed a statistically significant correlation with the otolaryngological evaluation and the results from the otoacoustic emissions in infants from zero to four months of age. The 1,000Hz test tone tympanometry was more sensitive to identify middle ear alterations and both test tones (226 and 1000Hz) were specific to identify the tympanograms of infants with OAEs.
